# Association between Multi-Domain Lifestyle and Objective Cognitive Impairment in Elderly People with SCD and MCI in Chinese Communities

**DOI:** 10.3390/healthcare12181879

**Published:** 2024-09-19

**Authors:** Yuqin Sun, Ruifen Zhang, Zhiqun Mao, Jiajun Yin, Yuanyuan Zhou, Yue Wu

**Affiliations:** 1 Department of Geriatric Psychiatry, the Affiliated Mental Health Center of Jiangnan University, Wuxi Central Rehabilitation Hospital, Wuxi 214151, China18956942757@163.com (R.Z.);; 2 Department of Nursing, Wuxi Medical College, Jiangnan University, 1800 Li Hu Avenue, Wuxi 214062, China; 3 Department of Medical Clinical Laboratory, the Affiliated Mental Health Center of Jiangnan University, Wuxi Central Rehabilitation Hospital, Wuxi 214151, China; 4 Department of Psychotherapy, the Affiliated Mental Health Center of Jiangnan University, Wuxi Central Rehabilitation Hospital, Wuxi 214151, China

**Keywords:** subjective cognitive decline, mild cognitive impairment, healthy lifestyle, cognitive function, correlation

## Abstract

Objectives: Controlling the lifestyle associated with dementia risk can delay the process of cognitive decline. Subjective cognitive decline (SCD) and mild cognitive impairment (MCI) are early states in the development of dementia and are also the window period for early intervention in dementia. The purpose of this study was to explore the association between multi-domain lifestyle and objective cognitive impairment in elderly people with SCD and MCI in Chinese communities and to provide reference for effective implementation of precise health management measures to reduce the risk of dementia. Methods: A total of 265 middle-aged and elderly volunteers recruited from the community were divided into SCD group (107 cases), MCI group (80 cases), and healthy control (HC) group (78 cases). All participants received clinical interview, examination, and cognitive assessments. Results: The total Dementia Risk Reduction Lifestyle Scale (DRRLS) scores in the HC, SCD, and MCI groups [110.00 (11.25) vs. 101.00 (10.00) vs. 79.50 (20.75)] exhibited statistically significant differences among them. The total score of the DRRLS showed a significant negative correlation with the Trail-Making Test (TMT), and significant positive correlations with both the Verbal Fluency Test (VFT) and Auditory Verbal Learning Test (AVLT) scores (*p* < 0.05). After adjusting for confounding factors, such as age and years of education, multiple linear regression analysis revealed several points. In the SCD group, brain-strengthening exercise and interpersonal relationship scores were negatively correlated with TMT scores (β = −11.257, −15.077; all *p* < 0.05), while health responsibility, smoking control behavior, and interpersonal relationship scores were positively correlated with AVLT scores (β = 0.485, 0.344, and 0.406; all *p* < 0.05). In the MCI Group, brain-strengthening exercise, brain-healthy diet, and interpersonal relationship were negatively correlated with TMT (β = −22.011, −16.206, −11.696; all *p* < 0.01), whereas health responsibility, mental activity, smoking control behavior, interpersonal relationship, and stress management were positively correlated with AVLT (β = 0.450, 0.435, 0.308, 0.256, 0.607; all *p* < 0.05). Conclusions: In Chinese communities, the unhealthy lifestyle of elderly individuals with SCD and MCI is significantly associated with cognitive function impairment. The greater their unhealthy lifestyle habits, the more pronounced the scope and severity of cognitive function impairment becomes. Furthermore, different dimensions of lifestyle have varying impacts on cognitive domains.

## 1. Introduction

In China, as the population ages, the cognitive decline and cognitive disorders among the elderly have become a significant public health concern. Alzheimer’s disease (AD) is a major chronic disease that poses a serious threat to the health and lives of the elderly. China is a large country with a large elderly population, and the number of people with AD has exceeded 15 million, ranking first in the world, and is expected to exceed 40 million by 2050 [1]. The burden of family and society caused by AD will be a severe challenge to the whole society and healthy development of China. It is predicted that over the next decade, the Crude Mortality Rate (CMR) for AD and other dementias in China will increase to 9.66 per 100,000 people, while the Age-Standardized Mortality Rate (ASMR) is expected to decrease to 3.42 per 100,000 people. The upward trend in CMR and downward trend in ASMR suggest the further development of population aging and dementia mortality in the future decades in China [2]. Due to the complexity of the etiology and pathological mechanisms, there is no effective cure for the dementia stage of AD relative to the rapidly growing number of patients, but early non-pharmacological interventions for target populations at risk of progressing to AD can slow or delay the development of dementia. Subjective cognitive decline (SCD) and mild cognitive impairment (MCI) are precursors to AD and are common in the elderly population, where they are two to four times more likely to progress to AD than in normal elderly people. SCD is defined as a subjective perception of significant cognitive decline in a person with a normal objective cognitive examination, but there may be mild impairment in complex neurocognitive domains. A study indicates that 50% to 80% of elderly individuals assessed at memory clinics report a decline in some cognitive functions [3]. MCI indicates mild cognitive impairment, but the impact on daily activities and social functioning has not yet reached the level of AD. Globally, the prevalence of MCI is approximately 19.7%, and this proportion has been increasing over time, particularly after 2019 [4]. Both SCD and MCI patients have complaints of cognitive decline of their own origin and are able to actively seek medical help, which is the best window for early intervention in AD [5]. The aim of this study was to determine the cognitive status of the older population by using a localized Quick Cognitive Screening Scale for the Elderly (QCCS-E) questionnaire [6].

Dementia prevention, intervention, and care: 2024 report of the Lancet standing Commission recently identified 14 potentially modifiable risk factors for AD, most of which are associated with poor lifestyle, such as hypertension, obesity, smoking, depression, lack of physical activity, social isolation, excessive alcohol consumption, and so on [7]. It is estimated that interventions targeting modifiable risk factors in the preclinical stage of the disease, through the adoption of healthy lifestyle and behavior habits, may prevent or delay 40% of AD cases [8]. In recent years, the incidence of AD in developed countries has been on a downward trend by helping the population abandon bad lifestyles and controlling vascular risk factors [9].

In recent years, China has been taking action to promote healthy aging, building a community network for elderly cognitive impairment management, and the concept of preventing AD by changing unhealthy lifestyles is increasingly being recognized [10,11]. Since the changes in different cognitive domains at the clinically early stage of AD are not synchronized, and the degree of brain function damage caused by different categories of unhealthy lifestyles is different, it is necessary to study the correlation between lifestyle and different cognitive domains in SCD and MCI patients, providing reference data for healthcare professionals to develop targeted health management measures for high-risk AD populations [12].

In recent years, in order to achieve healthy aging, the Chinese government has established a community health management network for the elderly with cognitive impairment to promote the concept and practice of AD prevention and treatment to change the unhealthy lifestyle. In the early clinical stage of AD, the changes in different cognitive domains are not synchronized, and the characteristics of brain function impairment caused by different types of adverse lifestyle are also different. This study took elderly people in Chinese communities as samples to investigate the association between multi-domain lifestyle and objective cognitive impairment in patients with SCD and MCI, ho-ping to effectively reduce the risk of dementia in elderly people in communities through precise multi-domain healthy life interventions.

## 2. Materials and Methods

### 2.1. Participants

A cross-sectional study was conducted in Wuxi, Jiangsu Province, China from October 2022 to June 2023. We recruited 300 volunteers aged 55 and above in the community through a questionnaire survey. The inclusion criteria were as follows: (1) age 55–80 years, primary school education and above; (2) having basic verbal expression and writing ability; (3) no serious physical illness affecting cognitive function; (4) voluntarily participated in this study and signed an informed consent form. The exclusion criteria were as follows: (1) significant cognitive impairment and severe mental illness; (2) any serious untreated systemic disease or unstable chronic somatic diseases. This study was approved by the Ethics Committee of Wuxi Mental Health Center (N0: WXMH-CIRB2021LLky001) and conformed to the Declaration of Helsinki. Stratified by sex and age, all participants were recruited directly by the researchers of this study. The purpose, risks, and benefits of the study and the principles of confidentiality and voluntary participation were explained to the 300 participants either orally or by text. Participants were allowed 1 week to consider whether to participate, and the final valid sample size was 265, all of whom signed the informed consent form.

### 2.2. Assessment and Grouping

#### 2.2.1. General Information Questionnaire

Sociodemographic information that could potentially influence the participants cognition was collected using a simple questionnaire designed by the researcher. General information questionnaire includes gender, age, level of education, marital status, occupational classification, living situation, smoking history, alcohol consumption, hypertension, diabetes, and body mass index (BMI).

#### 2.2.2. Subject Cognitive Assessment

Subjective cognitive symptoms were evaluated by the subjective cognitive decline questionnaire9 (SCD-Q9). It is a self-rating scale, including three dimensions, overall memory ability, daily activity ability, and time comparison, with nine entries and a total score of 0 to 9. Researchers conducted questionnaire assessments for the participants; the higher the score, the higher the likelihood of cognitive impairment. The questionnaire’s split-half reliability was 0.890, and the Cronbach’α coefficient was 0.880, indicating high reliability [13].

#### 2.2.3. Global Cognitive Evaluation

Global cognitive function was evaluated by the QCSS-E. It contains 51 items that assess 12 cognitive domains: immediate memory, naming of objects, verbal fluency, visuospatial ability, digit span, auditory imitation, visual imitation, abstract ability, command, delayed memory, simple calculation, and temporal and spatial orientation. The total score of the QCSS-E distributes from 0 to 85, and the lower the score, the worse the impairment of cognitive function. The scale retest reliability was 0.972, and the Cronbach’α coefficient was 0.814. The QCSS-E showed good reliability and validity among older Chinese adults [14].

#### 2.2.4. Dementia Risk Reduction Lifestyle Scale (DRRLS)

The status of healthy lifestyle was adopted using the DRRLS, developed by Zhang Jinying et al. It measures eight dimensions, health responsibility, brain-strengthening exercise, mental activity, brain-healthy diet, smoking control behavior, interpersonal relationship, stress management, and spiritual growth, with a total score of 32~128 points. Higher scores indicate that the lifestyle and behavioral habits of participants are more conducive to reducing the risk of dementia and promoting brain health. A scale with a split-half reliability of 0.909, re-test reliability of 0.864, and a Cronbach’α coefficient of 0.862 was used. Researchers assessed the quality of the dementia-risk lifestyles of participants based on their DRRLS scores [15].

#### 2.2.5. Core Neurocognitive Assessment

Objective cognitive function was assessed using core neurocognitive tests (CNT), including Trail-Making Test (TMT), Verbal Fluency Text (VFT), and Auditory Verbal Learning Test (AVLT). The Trail-Making Test-A (TMT-A) requires participants to connect numbers from 1 to 25 in ascending order as quickly as possible; the Trail-Making Test-B (TMT-B) involves numbers embedded within squares and circles, requiring participants to alternate between the two shapes while connecting the numbers in sequence, thereby measuring information processing speed and flexibility, respectively. In this study, the time taken to complete TMT-B is used as the scoring criterion; the longer the time, the more severe the impairment of executive function. The VFT recorded the total number of animal names correctly uttered by participants within 1 min, with 1 point for each name correct; the lower the score, the more severe the impairment of verbal function. The AVLT recorded the number of long-delayed memories correctly recalled for 12 words in 3 different categories, with 1 point for each word correct; the lower the score, the more severe the impairment of verbal function. The AVLT recorded the number of correct long-delay recalls of 12 words in three different categories, with 1 point per correct word, and the lower the score, the more severe the delayed memory impairment; the sums of the sensitivity and specificity of the three tests in identifying mild cognitive impairment were 1.67, 1.80, and 1.94, respectively [16,17,18,19]. All instruments used in this study have been previously validated for use in the an elderly Chinese population, ensuring the reliability and comparability of our findings with other studies.

### 2.3. Data Collection and Clinical Grouping

All volunteers who met the recruitment criteria were notified to come to the hospital to complete the general information survey, scale assessment, core neurocognitive tests, psychiatric examination, necessary auxiliary examination. Finally, 265 participants completed all the tests. Based on the examination results, participants were divided into three groups: HC, SCD, and MCI. The SCD group was formulated with reference to the concepts of the 2014 International Working Group: (1) presence of subjective cognitive complaints, SCD-Q9 > 3; (2) normal overall cognition, QCSS-E ≥ 75 [5]. The MCI group was formulated with reference to Petersen’s diagnostic criteria [20]: (1) presence of subjective cognitive complaints, SCD-Q9 > 3; (2) mildly impaired overall cognition, QCSS-E: 65~75. Healthy control group (HC): (1) no subjective cognitive complaints, SCD-Q9 ≤ 3; (2) normal overall cognition, QCSS-E > 80. According to the criteria, there were 78 cases in the HC group, 107 cases in the SCD group, and 80 cases in the MCI group.

### 2.4. Statistical Method

All statistical analyses were performed using SPSS26.0 statistical software (IBM Corporation., Armonk, NY, USA), R Studio (version 4.3.2), and GraphPad Prism version 9.3.5. Quantitative information conforming to normal distribution was described as mean ± standard deviation ($\bar{x}$ ± s). Skewed-distribution quantitative information was expressed as median (interquartile spacing) [M(OR)]. Counting variables were expressed in terms of frequency and percentage of ingredients. Comparisons of measurement and count data among the three groups were performed using the Wilcoxon rank sum test and the Chi-square test, respectively. Spearman correlation coefficient was used to analyze the correlation between DRRLS scores and CNT scores in SCD and MCI groups. The correlation results have undergone multiple comparison adjustments. The effects of multi-domain lifestyle on objective cognitive function were analyzed via multiple linear regression. The level of significance was set at *p* < 0.05.

## 3. Results

### 3.1. Comparison of Demographic Data and Clinical Features among the Three Groups

A total of 265 effective samples who met the inclusion criteria were finally obtained, including 126 males and 139 females, with an average age of 63.55 (±6.68) years. Among them, there were 78 healthy controls (HC group), 107 participants with SCD, and 80 participants with MCI. There were no significant differences in marital status, occupation classification, drinking, hypertension, or diabetes among the three groups, while there were significant differences in gender, age, level of education, living situation, smoking, BMI, QCSS-E, and SCD-Q9, as shown in Table 1.

### 3.2. Comparison of the Results of the DRRLS and CNT Test Scores among the Three Groups

As shown in Table 2, there were no statistically significant differences in the stress management (*p* = 0.057) and spiritual growth (*p* = 0.866) scores of the DRRLS among the three groups. However, the total score of DRRLS and other dimensions were higher in the HC group than in the SCD group and higher in the SCD group than in the MCI group. Moreover, the MCI group exhibited the highest score in stress management and the lowest score in mental activity among the eight dimensions of DRRLS. There were statistically significant differences in the core neuropsychological scores among the three groups (*p* < 0.001); the HC group had the highest VFT and AVLT scores.

### 3.3. Correlation Results Correlation Analyses between DRRLS Score and Neurocognitive Test Score in SCD and MCI Groups

**Figure 1:**
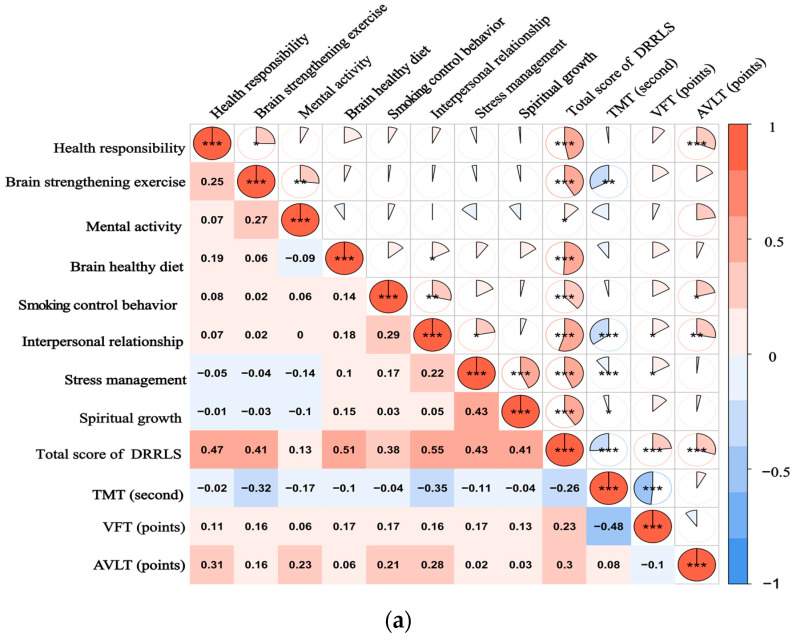

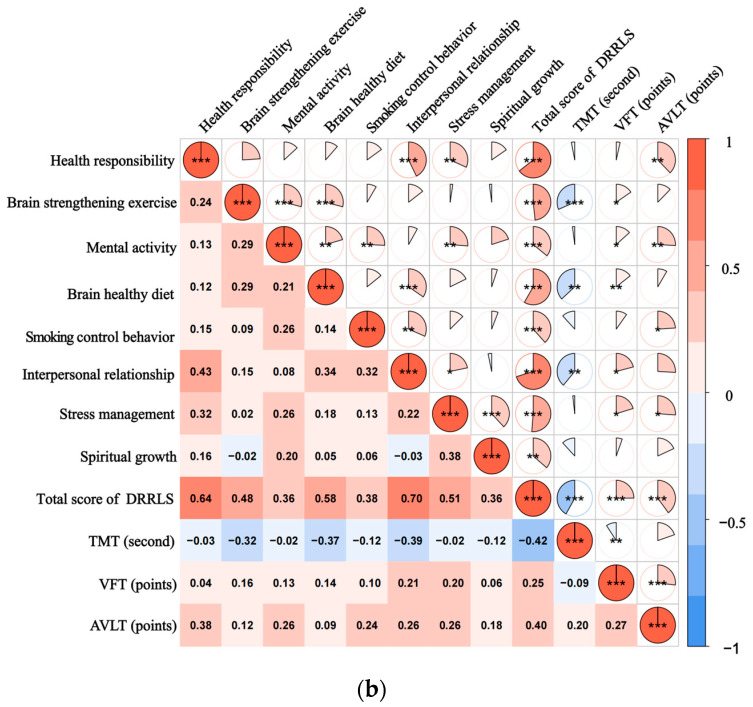
(**a**) illustrates the spearman correlation heatmap between the DRRLS and CNT scores among individuals with SCD; (**b**) presents a similar correlation heatmap for participants diagnosed with MCI. The color intensity within the heatmap reflects the strength and direction of the correlation coefficients, with a color gradient ranging from blue, indicating negative correlations, to red, indicating positive correlations. * *p*-Value ˂ 0.05, ** *p*-Value ˂ 0.01, and *** *p*-Value ˂ 0.001. A scale bar is provided at the bottom of the heatmap to represent the range of correlation values from −1 to 1.

The Spearman correlation analysis of DRRLS scores and neurocognitive scores in SCD and MCI groups showed that the total score of DRRLS was negatively correlated with TMT (r = −0.259, −0.418, *p* < 0.05) and positively correlated with VFT (r = 0.235, 0.248, *p* < 0.05) and AVLT (r = 0.295, 0.398, *p* < 0.01). Among the DRRLS subfields, brain-strengthening exercise, brain-healthy diet, and interpersonal relationship were negatively correlated with TMT (r = −0.315–(−0.389), *p* < 0.01), while health responsibility, mental activity, smoking control behavior, interpersonal relationship, and stress management were positively correlated with AVLT (r = 0.209–0.377, *p* < 0.05), as shown in Table 3 and Figure 1. The correlation results have been adjusted with a correction for multiple comparisons.

### 3.4. Multiple Linear Regression Analysis of Factors Influencing Patients with SCD and MCI

TMT, VFT, and AVLT scores were taken as dependent variables (assigned as measured values), and DRRLS factor scores and total scores with statistical significance in correlation analysis were taken as independent variables (assigned as measured values). After adjusting for confounding factors, such as age and years of education, multiple linear regression analysis was conducted. The results are shown in Table 4. In the SCD group, brain-strengthening exercise and interpersonal relationship were independently correlated with TMT (β = −11.257, −15.077, *p* < 0.05), and health responsibility, tobacco control behavior, and interpersonal relationship were independently correlated with AVLT (β = 0.485, 0.344, 0.406, *p* < 0.05). MCI Group: brain-strengthening exercise, brain-healthy diet, and interpersonal relationship were independently correlated with TMT (β = −22.011, −16.206, −11.696, *p* < 0.01), and health responsibility, mental activity, smoking control behavior, interpersonal relationship, and stress management were independently correlated with AVLT (β = 0.450, 0.435, 0.308, 0.256, 0.607, *p* < 0.05). The total DRRLS score was independently correlated with TMT, AVLT, and VFT in SCD and MCI patients (β = −1.253, 0.035, 0.070; −1.050, 0.058, 0.033, *p* < 0.01).

## 4. Discussion

AD has a covert onset, and the cognitive decline in the elderly is often regarded as a normal part of aging. By the time most patients seek medical attention, they are already in the middle to late stages of the disease, where irreversible brain damage has occurred, and the optimal window for treatment has been missed. SCD and MCI represent different stages in the pre-AD phase. Longitudinal observational studies have found that SCD occurs on average about 10 years before a dementia diagnosis, while MCI may progress to dementia within 5 to 7 years [21]. During these two stages, implementing diverse and effective interventions targeting modifiable risk factors can delay or slow down the onset of dementia.

The latest epidemiological survey data indicate that the prevalence rates of SCD and MCI in the elderly are 25.0~50.0% and 5.0~36.7%, respectively, representing a significant proportion of the population in the early stages of AD [22,23,24]. This study’s sample was derived from a community-dwelling elderly population, with the proportions of SCD and MCI being 40.4% and 30.2%, respectively, which are at a medium level. Although SCD and MCI share similar neuropathological patterns with AD, there are differences in the specific domains and severity of cognitive impairment [5]. Therefore, exploring the levels of lifestyle modifications that reduce the risk of dementia in SCD and MCI, as well as their association with cognitive deficits, can assist clinical practitioners in delivering personalized and precise health management strategies for different target populations.

This study demonstrates that the total score of DRRLS, as well as the scores of brain-strengthening exercise, brain-healthy diet, mental activity, health responsibility, interpersonal relationship, and smoking control behavior, were all significantly higher in the normal control group compared to both the SCD and MCI groups. It indicates that the health level of lifestyle and behavior habits of patients with pre-AD has been reduced in multiple dimensions, and primary healthcare personnel can carry out multi-field intervention in brain health from sports, nutrition, cognition, and other aspects service. The research findings both domestically and internationally also underscore the significance of cognitive engagement and cerebral stimulation in community health management, deeming it a primary preventive measure against dementia within the targeted population [25,26].

Although SCD is defined as the absence of objective cognitive impairment, most studies have concluded that there is mild cognitive impairment in SCD, and due to the compensatory effect of cognitive reserve, abnormalities are difficult to be detected by conventional cognitive tests at the individual level, and need to be captured by sensitive and complex neuropsychological tests above the group level [27].

In this study, the score of the core neuropsychological test of SCD was between the normal control and MCI patients, and the difference was statistically significant, indicating that executive function, language function, and delayed memory were changed in SCD stage. Compared with executive function and delayed memory, the impairment of language function was relatively slow with the progression of the disease. These results indicate that the changes in different cognitive domains in the early stage of AD are non-synchronous, which suggests that to accurately reduce the risk of dementia and delay the occurrence of dementia, health management measures with appropriate content and intensity should be formulated for different cognitive domains at different stages. The findings of the prevention of cognitive decline team in high-income countries suggest that undifferentiated prevention of cognitive decline is not sufficient, and that the cognitive benefits in the intervention mode mainly depend on the correct identification of the target population, the appropriate form and intensity of the intervention [28,29]. A healthy lifestyle and behavioral habits could lower the risk of dementia, and evidence-based interventions have become an important strategy for the primary prevention of AD [30]. This study showed that brain-strengthening exercise was independently associated with TMT in patients with SCD and MCI, indicating that insufficient physical activity has a negative impact on executive function. Several studies have shown that the prefrontal cortex is closely related to executive function, and moderate aerobic exercise can keep the hippocampus and prefrontal cortex regions of older adults flexible and responsive [31,32].

There is widespread lack of physical activity among the elderly in China, which may be related to traditional misconceptions. Indeed, most adverse events related to physical activities in the elderly stem from inappropriate exercise [33]. The China Geriatric Nursing Alliance advocates for brain-strengthening exercises as the primary lifestyle intervention for addressing the prevalence of AD. Furthermore, it is recommended that elderly individuals with SCD and MCI receive a tailored exercise prescription, including moderate aerobic exercise and comprehensive sports activities based on their individual physical condition [34,35]. The reserve hypothesis posits that maintaining positive interpersonal relationships in older adults serves as a beneficial stress stimulus, which can safeguard or enhance neuron growth [36]. Our findings demonstrate an independent association between interpersonal relationship and executive function, as well as delayed memory, in both individuals with SCD and MCI patients. Hirabayashi et al. reported that social isolation is linked to brain atrophy and cognitive decline, while exposing older adults to socially stimulating groups can potentially prevent or even reverse the decline in brain volume and improve cognitive function [37,38]. These collective findings underscore the significance of interventions aimed at ameliorating social isolation for dementia prevention and treatment [39]. It is recommended that communities actively engage in group interventions tailored for older adults, such as chess and card activities or virtual social interaction training utilizing internet technology.

Balanced dietary nutrition may affect the occurrence and development of AD through the “gut–brain” axis and epigenetic pathways. Studies have found that the microflora with anti-inflammatory activity in the gut of patients with MCI and AD decreased, inducing neuroinflammatory changes and affecting brain function [40,41]. We found that a brain-healthy diet was an influential factor in executive function in patients with MCI. Horie et al. reported that dietary intervention can significantly improve the executive function of patients with MCI [42]. In community services, healthcare workers should promote the importance of healthy eating habits for brain health and actively recommend the Mediterranean diet pattern [43]. Health responsibility and smoking control behavior were independently correlated with delayed memory in patients with SCD and MCI [44]. Health responsibility includes self-care awareness, ability to obtain and apply health information and services, etc. The stronger the sense of health responsibility, the better the individual’s ability to prevent dementia. Serper et al. reported that the health responsibility of the elderly is closely related to attention, computing ability, and memory, which is consistent with the results of this study [45]. Although the role of smoking in AD is still a controversial topic, long-term smoking can damage the endothelium of blood vessels, lead to arteriosclerosis, and trigger chronic cerebral ischemia. In addition, the high levels of carbon monoxide in cigarette smoke cause oxygen deprivation to the brain, both of which can lead to memory loss. The present study demonstrated a positive association between mental activity, stress management, and AVLT performance in patients with SCD and MCI. Additionally, these factors were independently associated with delayed memory in patients with MCI. Some studies have reported that cognitive training and decompression are beneficial to the overall cognitive function of the elderly in different life cycles, among which delayed memory and memory retention rate are the most significant improvements. The mechanism may be related to the activation of neuroplasticity in the dorsolateral prefrontal cortex and bilateral parietal cortex by cognitive training and the increase in gray matter volume in the hippocampus and cingulate gyrus by self-relaxation and decompression [46,47,48].

Due to the cross-sectional nature of this comparative study, certain limitations should be noted. Firstly, causal inference cannot be definitively drawn from the study results. Secondly, potential confounding factors, such as AD risk genes or other somatic diseases, may impact our findings. Additionally, our sample, although reasonably sized, is derived from a specific geographical area and may not be fully representative of the broader population, potentially affecting the accuracy of our data. Future research should aim to address these limitations by employing longitudinal study designs to better understand the causal relationships between lifestyle factors and cognitive health. Additionally, studies with larger and more diverse samples, incorporating both objective measures of lifestyle behaviors and a broader range of cognitive assessments, would enhance the validity and generalizability of the findings.

## 5. Conclusions

The unhealthy lifestyle of elderly individuals with SCD and MCI in Chinese communities is closely associated with objective cognitive impairment, and various types of lifestyles exert distinct effects on the domain and extent of cognitive function. We recommend that personalized and targeted interventions be developed based on assessment results when implementing lifestyle modifications for the prevention and treatment of AD.

Given the heterogeneity of lifestyle factors and their distinct influences on cognitive domains, we advocate for the development of personalized interventions that are informed by comprehensive assessments. These interventions should be multimodal, encompassing physical exercise interventions, nutritional therapy, psychosocial interventions, raising health awareness, etc. Emphasis is placed on the customization of healthcare based on individual differences in genetics, environment, and lifestyle. In terms of future research, longitudinal studies are warranted to assess the long-term impact of such interventions on the cognitive trajectories of individuals with SCD and MCI [49]. In the future, we can rely on digital means, such as digital detection and prevention methods, and various digital technologies were independently used by people aged 50 years or older for health promotion and disease prevention [50]. Healthcare professionals can use internet platforms to enhance the information support for cognitive decline, provide accurate and effective disease knowledge, and pay attention to the cognitive follow-up for individuals with multiple risk factors [51].

## Figures and Tables

**Table 1:** Comparison of demographic characteristics and clinical data between the three groups (*n* = 265).

Variable	HC Group (*n* = 78)	SCD Group (*n* = 107)	MCI Group (*n* = 80)	χ^2^/Z	*p*-Value
Gender, *n* (%)				6.698	**0.035**
Male	46 (59.0)	49 (45.8)	31 (38.8)		
Female	32 (41.0)	58 (54.2)	49 (61.2)		
Age (years), *n* (%)				9.828	**0.043**
55–64	50 (64.1)	57 (53.3)	33 (41.2)		
65–74	25 (32.1)	44 (41.1)	38 (47.5)		
75–84	3 (3.8)	6 (5.6)	9 (11.2)		
Level of education, *n* (%)				31.794	**<0.001**
Primary school	10 (12.8)	27 (25.2)	37 (46.2)		
Junior high school	39 (50.0)	63 (58.9)	31 (38.8)		
High school and above	29 (37.2)	17 (15.9)	12 (15.0)		
Marital status, *n* (%)				4.004	0.135
Single	73 (93.6)	96 (89.7)	67 (83.8)		
Married	5 (6.4)	11 (10.3)	13 (16.2)		
Occupational classification, *n* (%)				4.418	0.110
Mental labor	18 (23.1)	23 (21.5)	9 (11.2)		
Physical labor	60 (76.9)	84 (78.5)	71 (88.8)		
Living situation, *n* (%)				7.669	**0.022**
Living alone	6 (7.70)	9 (8.4)	16 (20.0)		
Not living alone	72 (92.3)	98 (91.6)	64 (80.0)		
Smoking, *n* (%)				11.621	**0.020**
Smoking	14 (17.9)	18 (16.8)	24 (30.0)		
Non-smoker	57 (73.1)	86 (80.4)	55 (68.8)		
Quit Smoking	7 (9.0)	3 (2.8)	1(1.2)		
Drinking, *n* (%)				6.071	0.194
None	60 (76.9)	84 (78.5)	65 (81.2)		
Occasional (1 time/week)	9 (11.5)	17 (15.9)	13 (16.2)		
Regular (3 time/week)	9 (11.5)	6 (5.6)	2 (2.5)		
Hypertension, *n* (%)				3.930	0.140
Yes	22 (28.2)	45 (42.1)	27 (33.8)		
No	56 (71.8)	62 (57.9)	53 (66.2)		
Diabetes, *n* (%)				1.947	0.378
Yes	4 (5.1)	9 (8.4)	9 (11.2)		
No	74 (94.9)	98 (91.6)	71 (88.8)		
BMI (kg/m^2^), M (OR)	22.99 (4.49)	24.30 (2.29)	25.86 (3.33)	53.632	**<0.001**
QCSS-E (points), M (OR)	82.00 (1.00)	76.00 (2.00)	71.00 (4.00)	236.185	**<0.001**
SCD-Q9 (points), M (OR)	2.00 (1.50)	5.00 (2.50)	5.50 (3.40)	69.482	**<0.001**

Notes: Values are presented as the median (interquartile spacing) for continuous variables and *n* (%) for categorical variables. The bold *p*-values (<0.05) represent significant difference between groups, with regard to a domain score. Abbreviation: HC, normal cognition; SCD, subjective cognitive decline; MCI, mild cognitive impairment; M (OR), median (interquartile spacing); Z, Wilcoxon rank sum; χ^2^, Chi-square test; BMI, body mass index; QCSS-E, Quick Cognitive Screening Scale for the Elderly; SCD-Q9, subjective cognitive decline questionnaire9.

**Table 2:** Comparison of DRRLS and CNT test results in three groups.

Variable	HC Group (*n* = 78)	SCD Group (*n* = 107)	MCI Group (*n* = 80)	Z	*p*-Value
DRRLS					
Health responsibility, M (OR)	3.75 (0.50)	3.50 (0.75)	2.38 (1.50)	52.944	**<0.001**
Brain-strengthening exercise, M (OR)	3.40 (1.20)	2.80 (0.60)	1.80 (0.80)	108.772	**<0.001**
Mental activity, M (OR)	3.50 (1.13)	2.00 (1.00)	1.50 (1.00)	86.617	**<0.001**
Brain-healthy diet, M (OR)	3.60 (0.65)	3.40 (0.60)	2.20 (1.15)	108.704	**<0.001**
Smoking control behavior, M (OR)	2.50 (0.50)	2.50 (1.00)	1.75 (1.50)	22.186	**<0.001**
Interpersonal relationship, M (OR)	3.80 (0.40)	3.60 (1.00)	2.20 (1.75)	61.473	**<0.001**
Stress management, M (OR)	3.25 (1.00)	3.25 (1.00)	3.25 (0.75)	5.714	0.057
Spiritual growth, M (OR)	3.20 (1.05)	3.40 (1.20)	3.20 (1.20)	0.288	0.866
Total score of DRRLS, M (OR)	110.00 (11.25)	101.00 (10.00)	79.50 (20.75)	132.889	**<0.001**
TMT (second), M (OR)	125.50 (28.00)	143.00 (12.00)	174.00 (27.00)	72.069	**<0.001**
VFT (points), M (OR)	19.00 (3.00)	15.00 (2.00)	11.00 (2.00)	157.298	**<0.001**
AVLT (points), M (OR)	5.00 (1.00)	3.00 (1.00)	2.00 (1.00)	118.946	**<0.001**

Notes: Values are presented as the median (interquartile spacing) for continuous variables. The bold *p*-values (<0.05) represent significant difference between groups with regard to a domain score. Abbreviation: M (OR), median (interquartile spacing); Z, Wilcoxon rank sum; DRRLS, Dementia Risk Reduction Lifestyle Scale; TMT, Trail-Making Test; VFT, Verbal Fluency Text; AVLT, Auditory Verbal Learning Test.

**Table 3:** Correlation analysis of DRRLS total and dimensional scores with core neurocognitive test in patients in the SCD and MCI groups (*r*—value).

Project	SCD			MCI		
	TMT	VFT	AVLT	TMT	VFT	AVLT
Health responsibility	−0.020	0.110	0.311 **	−0.031	0.035	0.377 **
Brain-strengthening exercise	−0.325 **	0.161	0.162	−0.315 **	0.155	0.124
Mental activity	−0.173	0.060	0.226 *	−0.021	0.134	0.259 *
Brain-healthy diet	−0.102	0.171	0.061	−0.368 **	0.145	0.085
Smoking control behavior	−0.037	0.173	0.209 *	−0.119	0.099	0.240 *
Interpersonal relationship	−0.348 **	0.165	0.278 **	−0.389 **	0.210	0.259 *
Stress management	−0.111	0.172	0.019	−0.018	0.203	0.261 *
Spiritual growth	−0.044	0.132	0.034	−0.120	0.055	0.182
Total score of DRRLS	−0.259 *	0.235 *	0.295 **	−0.418 **	0.248 *	0.398 **

Notes: Employing Spearman correlation analysis. The bold *p*-values (<0.05) represent significant difference between groups with regard to a domain score. * *p*-Value < 0.05, ** *p*-Value < 0.01. Abbreviation: SCD, subjective cognitive decline; MCI, mild cognitive impairment; DRRLS, Dementia Risk Reduction Lifestyle Scale; TMT, Trail-Making Test; VFT, Verbal Fluency Text; AVLT, Auditory Verbal Learning Test.

**Table 4:** Results of Multiple Linear Regression Analysis of Factors Influencing Cognitive Function in Patients with SCD and MCI.

Dependent Variable	Variables Included in the Model	β	SE	β′	t	*p*-Value
SCD						
TMT	Brain-strengthening exercise	−11.257	4.368	−0.255	−2.577	**0.011**
	Interpersonal relationship	−15.077	2.797	−4.74	−5.391	**<0.001**
AVLT	Health responsibility	0.485	0.147	0.313	3.292	**0.001**
	Mental activity	0.173	0.119	0.146	1.456	0.148
	Smoking control behavior	0.344	0.140	0.233	2.451	**0.016**
	Interpersonal relationship	0.406	0.126	0.301	3.219	**0.002**
MCI						
TMT	Brain-strengthening exercise	−22.011	4.845	−0.462	−4.543	**<0.001**
	Brain-healthy diet	−16.206	5.169	−0.337	−3.135	**0.002**
	Interpersonal relationship	−11.696	4.055	−0.317	−2.885	**0.005**
AVLT	Health responsibility	0.450	0.130	0.369	3.466	**0.001**
	Mental activity	0.435	0.164	0.292	2.657	**0.010**
	Smoking control behavior	0.308	0.152	0.221	2.020	**0.047**
	Interpersonal relationship	0.256	0.125	0.228	2.050	**0.044**
	Stress management	0.607	0.214	0.305	2.840	**0.006**

Notes: The bold *p*-values (<0.05) represent significant difference between groups with regard to a domain score. Abbreviation: β, unstandardized coefficient; β′, standardized coefficient; SE, standard error; t, independent *t*-test; SCD, subjective cognitive decline; MCI, mild cognitive impairment; DRRLS, Dementia Risk Reduction Lifestyle Scale; TMT, Trail-Making Test; VFT, Verbal Fluency Text; AVLT, Auditory Verbal Learning Test.

## Data Availability

The data presented in this study are available on request from the corresponding author. The data are not publicly available due to privacy.
